# Older adults Care During a Time of Crisis: Israel’s Experience During Wartime

**DOI:** 10.1093/geroni/igaf122.4166

**Published:** 2025-12-31

**Authors:** Yakir Rottenberg, Shelley A Sternberg

**Affiliations:** Faculty of Medicine, Hebrew University of Jerusalem, Israel, Jerusalem, Yerushalayim, Israel; Maccabi Healthcare Services, Tel Aviv Israel, Tel Aviv, Tel Aviv, Israel

## Abstract

In recent decades, global crises like the COVID-19 pandemic and natural disasters have significantly challenged the ability to provide care for older adults, highlighting a critical research priority. Israel, with its advanced system of care for older adults and strong community support, performed well during COVID-19. This report describes efforts to maintain care during the June 2025 Israel-Iran war, a period of physical threat, disrupted medical services, psychological distress, and weakened social supports due to displacement and infrastructure damage. We highlight innovative strategies ensuring continuity of medical, mental health, and essential social services for older adults. Israel’s approach to emergency care for older adults rested on three pillars: (i) Leveraging past experience: Protocols from previous emergencies, including COVID-19, were quickly activated. This ensured continuity of care for older adults, especially those evacuated or in unsafe apartments, balancing safety with independence. (ii) Coordinated communication: Ongoing national and municipal collaboration and communication about at-risk older adults, enabled effective and timely, targeted interventions. (iii) Volunteerism and Civil Initiatives: A massive surge of community-led volunteer efforts provided transport, medications, and evacuee assistance, critically addressing urgent needs beyond government capacity. In conclusion, these three pillars – rapid response based on prior experience, a strong national and municipal system of communication and collaboration, and robust volunteerism – enabled continuous, high-quality care for older adults during this major crisis., These lessons can inform other systems aiming to enhance their resilience and capacity to provide care to older adults during emergency situations.

